# Neutral and functionally important genes shed light on phylogeography and the history of high‐altitude colonization in a widespread New World duck

**DOI:** 10.1002/ece3.4108

**Published:** 2018-06-04

**Authors:** Maria Lozano‐Jaramillo, Kevin G. McCracken, Carlos Daniel Cadena

**Affiliations:** ^1^ Laboratorio de Biología Evolutiva de Vertebrados Departamento de Ciencias Biológicas Universidad de Los Andes Bogotá Colombia; ^2^ Wageningen University & Research Animal Breeding and Genomics Wageningen The Netherlands; ^3^ Department of Biology University of Miami Coral Gables Florida; ^4^ Rosenstiel School of Marine and Atmospheric Sciences University of Miami Miami Florida; ^5^ Human Genetics and Genomics Hussman Institute for Human Genomics University of Miami Miller School of Medicine Miami Florida; ^6^ Institute of Arctic Biology and University of Alaska Museum University of Alaska Fairbanks Fairbanks Alaska

**Keywords:** adaptation, hypoxia, migration, natural selection

## Abstract

Phylogeographic studies often infer historical demographic processes underlying species distributions based on patterns of neutral genetic variation, but spatial variation in functionally important genes can provide additional insights about biogeographic history allowing for inferences about the potential role of adaptation in geographic range evolution. Integrating data from neutral markers and genes involved in oxygen (O_2_)‐transport physiology, we test historical hypotheses about colonization and gene flow across low‐ and high‐altitude regions in the Ruddy Duck (*Oxyura jamaicensis*), a widely distributed species in the New World. Using multilocus analyses that for the first time include populations from the Colombian Andes, we also examined the hypothesis that Ruddy Duck populations from northern South America are of hybrid origin. We found that neutral and functional genes appear to have moved into the Colombian Andes from both North America and southern South America, and that high‐altitude Colombian populations do not exhibit evidence of adaptation to hypoxia in hemoglobin genes. Therefore, the biogeographic history of Ruddy Ducks is likely more complex than previously inferred. Our new data raise questions about the hypothesis that adaptation via natural selection to high‐altitude conditions through amino acid replacements in the hemoglobin protein allowed Ruddy Ducks to disperse south along the high Andes into southern South America. The existence of shared genetic variation with populations from both North America and southern South America as well as private alleles suggests that the Colombian population of Ruddy Ducks may be of old hybrid origin. This study illustrates the breadth of inferences one can make by combining data from nuclear and functionally important loci in phylogeography, and underscores the importance of complete range‐wide sampling to study species history in complex landscapes.

## 1 INTRODUCTION

Phylogeographic analyses aimed at understanding historical processes underlying species distributions typically focus on associations between geography and the distribution of neutral genetic variation (Avise, 2000; Hickerson et al., 2010; Knowles, 2004). However, genes of functional importance can also be employed to investigate the history of species, particularly when populations are exposed to distinct environments with contrasting selective pressures (Deagle, Jones, Absher, Kingsley, & Reimchen, 2013; Feldman, Brodie, & Pfrender, 2009; Hoekstra, Drumm, & Nachman, 2004; Savage & Zamudio, 2016; Sork et al., 2016). Analyses of functionally important genes allow one to address questions about evolutionary processes such as adaptation and natural selection, complementing inferences of demographic processes based on patterns of geographic variation in neutral genes (Zamudio, Bell, & Mason, 2016). Here, we integrate analyses of neutral markers and protein‐coding genes potentially under selection to test hypotheses about the biogeographic and evolutionary history of a widespread species of duck with a broad latitudinal and elevational distribution in the New World.

A long‐standing question in biogeography is that of the evolutionary origin of high‐elevation organisms. In the New World, in particular, researchers have long been interested in understanding the origins of biotas occurring in the high Andes of South America, with a leading hypothesis being that tropical high‐elevation species are derived from low‐elevation ancestors occupying areas with similarly cool climates in the temperate zone (Chapman, 1917; Vuilleumier, 1986b). Examples of plant and animal lineages colonizing the high Andes from temperate regions and subsequently diversifying in tropical montane areas are consistent with the hypothesis that such lineages were preadapted to occur in cool high‐elevation environments and that this allowed them to occupy newly formed habitats resulting from the Andean uplift (Hughes & Eastwood, 2006; Sanín et al., 2009). However, because life at high elevations requires not only dealing with low temperatures but also coping with hypoxia, colonization of montane environments by species from low‐elevation areas may have also entailed adaptive evolution in traits related to oxygen (O_2_)‐transport physiology.

High‐elevation environments are of particular interest for research at the interface of biogeography, adaptation, and physiology because hypoxia increases markedly along elevational gradients, likely imposing strong selective pressures and setting limits to species ranges. In high‐elevation regions above 4,000 m, such as the high Andes, the partial pressure of oxygen is approximately 60% of that at sea level (Beall, 2006; Hopkins & Powell, 2001). Under such conditions, oxygen transport to tissues is reduced, altering metabolism and inhibiting physical activity (Hopkins & Powell, 2001; Storz & Moriyama, 2008). A variety of physiological compensatory mechanisms have evolved in high‐altitude organisms across the oxygen transport cascade, examples of which include both genetic adaptations and environmentally induced plasticity (Storz, 2010). One of the best‐studied examples of genetically based adaptation involves the blood O_2_ transport protein hemoglobin. Many organisms native to high altitude exhibit hemoglobin with a high affinity for oxygen (Weber, 2007); in birds, increased hemoglobin‐oxygen affinity has evolved convergently across multiple lineages occurring at high elevations (Natarajan et al., 2016). Studies of hemoglobin have also revealed environmentally induced phenotypic plasticity in response to pressures associated with high elevations. Unlike many high‐altitude endemics, which often maintain relatively low hemoglobin concentrations, species native to low altitude typically increase their hemoglobin concentration and associated hematocrit (i.e., the volume of packed red blood cells) upon ascending to high altitude, thereby increasing their total blood oxygen carry capacity (Lui et al., 2015; Monge & Leon‐Velarde, 1991; Tufts et al., 2013). Although there is evidence indicating that such response may be maladaptive over the long term due to increased blood viscosity (Storz & Moriyama, 2008; Storz, Scott, & Cheviron, 2010), over the short term, this plasticity is an essential component of the acclimatization response of many low‐altitude organisms.

Analyses of geographic variation in genes involved in O_2_ transport have provided important insights about the history of occupation of high‐elevation environments by birds. For example, estimates of gene flow between lowland and highland populations of Crested Ducks (*Lophonetta specularioides*) obtained based on hemoglobin alleles are much lower than those inferred from neutral markers; in light of biochemical properties of amino acid substitutions differing across elevations, such differences in hemoglobin gene flow are consistent with a role for selection structuring genetic and phenotypic variation (Bulgarella et al., 2012). Likewise, hemoglobin variants with increased affinity for oxygen occur at a greater frequency in high‐elevation populations of House Wrens (*Troglodytes aedon*) relative to those from lowland areas, and *F*_st_ across elevations for a functionally important site in the βA‐globin gene is greater than *F*_st_ values for most coding SNPs across the genome (Galen et al., 2015). Because the high‐elevation βA‐globin genotype is derived, colonization of high‐elevation environments by House Wrens presumably involved adaptive evolution in protein function.

**Figure 1:**
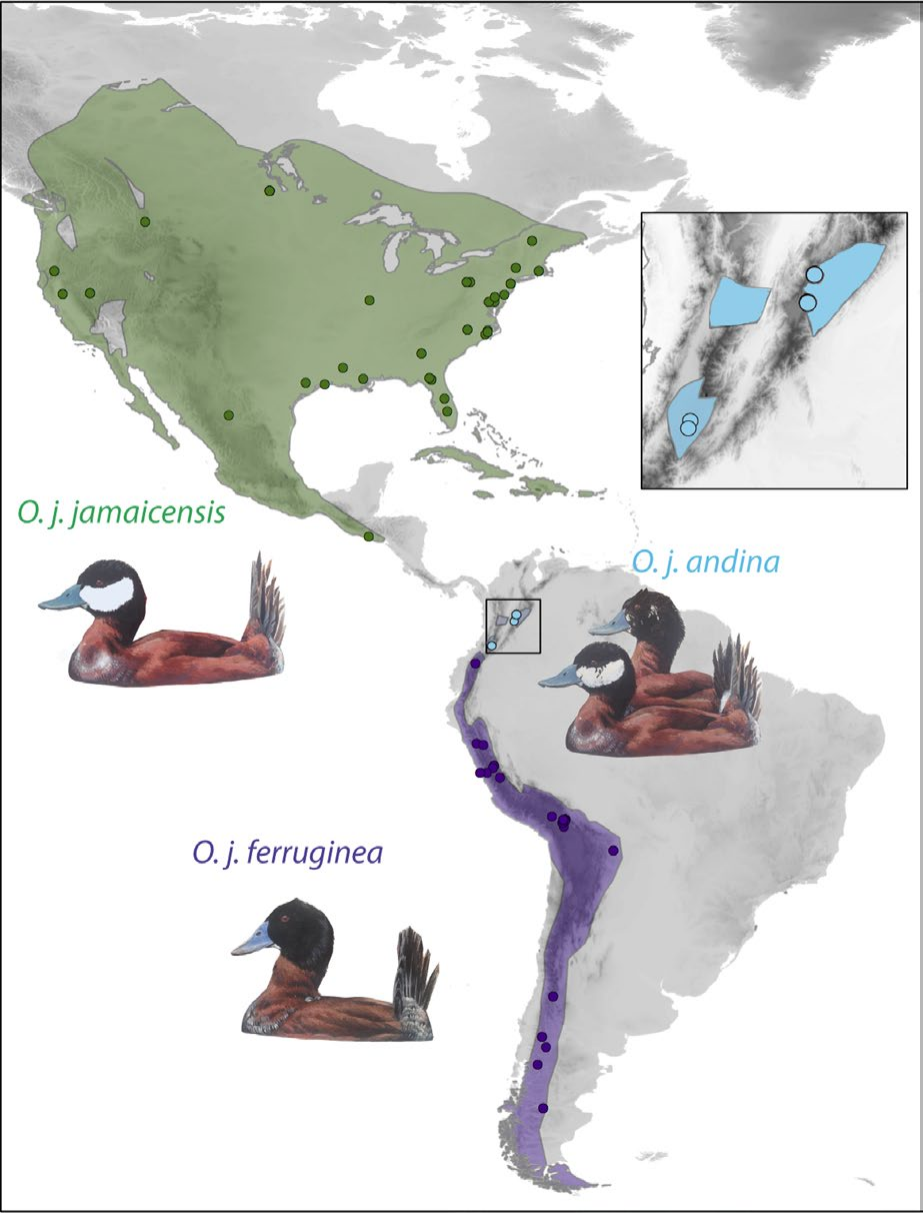
Current geographic distribution of Ruddy Ducks and locations where samples were obtained for our phylogeographic analyses

A case in which adaptive evolution of hemoglobins has been hypothesized to play a role in the evolutionary history of a species and facilitated its spread across landscapes is that of the Ruddy Duck (Anatidae, *Oxyura jamaicensis*), a diving duck found throughout the New World from southern Canada to Tierra del Fuego (Figure 1). Three subspecies are recognized based on facial plumage of males (Adams & Slavid, 1984; Fjeldså, 1986; Siegfried, 1976). The nominate North American subspecies (*O. j. jamaicensis*; all‐white cheek) occurs in North America and Middle America, the Colombian subspecies (*O. j. andina*; variable black‐and‐white cheek) is restricted to middle and high elevations in the Colombian Andes, and the Andean‐Patagonian subspecies (*O. j. ferruginea*; all back head) ranges throughout the Northern Andes and Southern Andes from southern Colombia to Tierra del Fuego. Consistent with the general hypothesis that highland tropical lineages are derived from temperate‐zone lineages and with a biogeographic hypothesis for Ruddy Ducks proposed by Fjeldså (1986), phylogeographic analyses using sequences of mitochondrial DNA, autosomal introns, and hemoglobin genes indicated that Ruddy Ducks dispersed from North America to the tropical Andes and subsequently to the southern Andes (Muñoz‐Fuentes, Cortazar‐Chinarro, Lozano‐Jaramillo, & McCracken, 2013). Furthermore, based on geographic variation in substitutions in the βA globin gene, it was proposed that, upon expanding its range, the species first acclimatized or adapted to highlands of the Andes (Muñoz‐Fuentes et al., 2013). A substitution putatively increasing the affinity of hemoglobin for oxygen (McCracken, Barger, Bulgarella, Johnson, Sonsthagen, et al., 2009) may have allowed Ruddy Ducks to establish in the Northern Andes and to spread southward along highland environments; as the species colonized precordilleran steppe habitats in southern South America, its hemoglobin seemingly adapted again to lowland conditions (Muñoz‐Fuentes et al., 2013). These inferences, however, were made based on molecular data sets that did not include population‐level sampling in the Northern Andes and were not validated by functional analyses. In fact, a subsequent analysis revealed that substitutions occurring in high‐elevation Ruddy Ducks have no discernible effects on the oxygen affinity of hemoglobin (Natarajan et al., 2015). Although this analysis did not rule out the possibility that genetic differences between lowland and highland Ruddy Ducks may affect other structural or functional properties of hemoglobins (Natarajan et al., 2015), it implies that the previously proposed biogeographic and evolutionary scenario (Muñoz‐Fuentes et al., 2013) may need reconsideration.

Here, we revisit the phylogeography of Ruddy Ducks and reexamine the potential role of adaptive evolution of hemoglobin genes in the establishment of its geographic range using multilocus analyses including data from both neutral and functional loci that for the first time involve dense sampling from the Colombian Andes. Our sampling scheme also allowed us to address the question of whether the Colombian population of Ruddy Ducks may be of hybrid origin as suggested by its wide variation in male facial plumage (Fjeldså, 1986; McCracken & Sorenson, 2005; Siegfried, 1976). Our extended sampling suggests that there is likely more complexity to biogeographic and evolutionary scenarios proposed by earlier studies.

## 2 METHODS

### 2.1 Sampling

To complete the geographic sampling of Ruddy Ducks employed in previous analyses, which only considered a handful of specimens from the Cordillera Central of the Colombian Andes (McCracken & Sorenson, 2005), we obtained 26 additional samples from the Cordillera Oriental of Colombia. These included (1) tissue samples from six specimens from Lake Fúquene (2,580 m), Cundinamarca, obtained from the Museo de Historia Natural at Universidad de los Andes, and (2) 20 blood samples we collected from Ruddy Ducks captured and released at two adjacent artificial lakes (i.e., abandoned gravel pits) located near the town of Guasca, Cundinamarca (2,630–2,640 m elevation; see Table S1).

### 2.2 PCR and DNA sequencing

Genomic DNA was extracted using a phenol/chloroform protocol (Sambrook & Russell, 2001). For each individual, we amplified and sequenced a total of seven genes using PCR protocols and primers (see Table S2) as described by Muñoz‐Fuentes et al. (2013). Loci examined were (1) the mitochondrial control region (578 bp); (2) four autosomal nuclear introns including phosphoenolpyruvate carboxykinase intron 9 (PCK1‐9; 316 bp), *N‐*methyl‐d‐aspartate‐1‐glutamate receptor intron 11 (GRIN1‐11; 308 bp), beta fibrinogen intron 7 (FGB‐7; 248 bp), and ornithine decarboxylase intron 5 (ODC1‐5; 345 bp); (3) the complete coding sequence of the α^A^‐globin subunit; and (d) exon 1, intron 1, exon 2, and part of intron 2 of the β^A^‐globin subunit (581 bp), encompassing the three previously described amino acid polymorphisms at positions 13, 14, and 69 documented by McCracken, Barger, Bulgarella, Johnson, Sonsthagen, et al. (2009). Sequences were edited using Geneious Pro 4.8.3 (http://www.geneious.com) or Sequencher v.4.6 (Gene Codes, Ann Arbor, MI) and aligned by eye using Se‐Al v.2.0a11 (Rambaut, 2002). The sequences generated were deposited in GenBank (accession numbers MH351796‐ MH352119).

### 2.3 Statistical analyses

To resolve the gametic phase of nuclear genes, we used PHASE v.2.1 (Stephens, Smith, & Donnelly, 2001); as with the previously published analysis, we used default settings of 1,000 burn‐in and 1,000 sampling iterations (‐×10), applying the algorithm five times consecutively (‐×5) for each locus with different random number seeds. Most genes showed high posterior allele pair probabilities (*p* > .99); for GRIN1 two individuals had allele pairs with *p* = .70 and two other individuals with *p* = .85. For PCK1, four individuals had allele pairs with *p* = .61.

We combined our new sequence data with previously published sequences for a total of 69 individuals of *O. j. jamaicensis* (North American lowlands), 31 of *O. j. andina* (Colombian highlands; only mtDNA data were available for five samples), and 42 of *O. j. ferruginea* (lowlands and highlands from Ecuador to Argentina). For simplicity, we refer hereafter to our three populations based on the regional distribution of the three Ruddy Duck subspecies: North America corresponds to areas occupied by *O. j. jamaicensis*, Colombia to areas occupied by *O. j. andina,* and Southern Andes to areas occupied by *O. j. ferruginea*. To infer relationships among haplotypes at each locus, we constructed unrooted parsimony allelic networks in TCS v1.21 (Clement, Posada, & Crandall, 2000). Because sample sizes differed among populations, we calculated allelic richness standardized to the smallest sample size using the Rarefaction Calculator (http://www2.biology.ualberta.ca/jbrzusto/rarefact.php).

To examine the distribution of genetic variation within and among North America, Colombia, and the Southern Andes, we calculated pairwise Φ_st_ values for each locus based on the Tamura & Nei (1993) nucleotide substitution model in Arlequin v.3.5.1.3 (Excoffier & Lischer, 2010). To determine whether different geographic populations (North America, Colombia, Southern Andes) are genetically distinct clusters, we used the multilocus clustering algorithm in the program STRUCTURE v 2.2.3 (Pritchard, Stephens, & Donnelly, 2000). We based this analysis on our seven‐locus data set, where each locus was coded as a haplotype, and included all the individuals for which no more than two of the loci were missing (*n* = 85 individuals). We used the admixture model (α = 1) with correlated allele frequencies and tested models for varying numbers of populations (*K *=* *2–4). Each analysis was run for one million generations (burn‐in 100,000 generations) and replicated 10 times. To choose the best value of *K,* we used the Evanno, Regnaut, and Goudet (2005) method. The outputs of the repeated runs at each *K* were summarized using the greedy algorithm in CLUMPP v1.1.2 (Jakobsson & Rosenberg, 2007).

### 2.4 Coalescent analyses of migration

To estimate gene flow (*M = m/u*) between populations, we used a population genetic approach based on isolation‐with‐migration coalescent models implemented in the program IMa2 (Hey, 2010). Using a two‐population model (i.e., multiple pairwise comparisons), this analysis allowed us to examine the direction of gene exchange based on estimates of historical gene flow between the three populations (Hey, 2005, 2010; Hey & Nielsen, 2004; Nielsen & Wakeley, 2001). Because IMa2 assumes no intralocus recombination, we tested for evidence of recombination in our data using the four‐gamete test (Hudson & Kaplan, 1985) in DnaSP v.5.10 (Librado & Rozas, 2009). GRIN1 and β^A^ showed evidence of recombination; for the former, we excluded five individuals following Muñoz‐Fuentes et al. (2013) and for the latter, we removed the recombinant blocks by eye. Because mtDNA is maternally inherited, we used an inheritance scalar of 0.25 and used the HKY mutation model for this locus (Hasegawa, Kishino, & Yano, 1985). For biparentally inherited nuclear genes, we used an inheritance scalar of 1.0 and implemented the infinite alleles model (Kimura, 1969). Because mtDNA and β^A^ may be under selection associated with the occupation of high‐elevation environments, we treated these two genes and the other four nuclear loci separately, and also ran the analysis with all genes combined. IMa2 was initially run with wide priors to explore the sensitivity of the parameters to varying upper bounds. These analyses were then repeated with uniform priors including the entire posterior distribution of each parameter observed in preliminary runs. For each run, we reported values of gene flow corresponding to the 90% highest posterior density (HPD) estimated by IMa2. To ensure that all the parameters converged, autocorrelation and effective sample sizes (ESS) were monitored, and runs were continued until the ESS for each parameter was at least 100.

## 3 RESULTS

Genetic structure between populations of Ruddy Ducks varied considerably across the seven loci, with Φ_st_ values ranging from 0.12 to 0.61 (Table 1). Despite this variation, genetic differentiation was significant for all population comparisons and loci, except between the Southern Andes and Colombia for the FGB intron.

**Table 1:** Φ_st_ values calculated for pairwise comparisons between three populations of Ruddy Ducks for seven loci. Bold values are significant at the 0.05 level

	Control region (mtDNA)	FGB	GRIN1	ODC1	PCK1	α^A^ hemoglobin	β^A^ hemoglobin
North America vs Colombia	**0.26**	**0.12**	**0.52**	**0.06**	**0.32**	**0.04**	**0.19**
Southern Andes vs Colombia	**0.31**	0.01	**0.81**	**0.47**	**0.18**	**0.20**	**0.37**
North America vs Southern Andes	**0.36**	**0.13**	**0.44**	**0.63**	**0.27**	**0.19**	**0.65**
Overall	**0.32**	**0.12**	**0.61**	**0.41**	**0.28**	**0.16**	**0.42**

**Figure 2:**
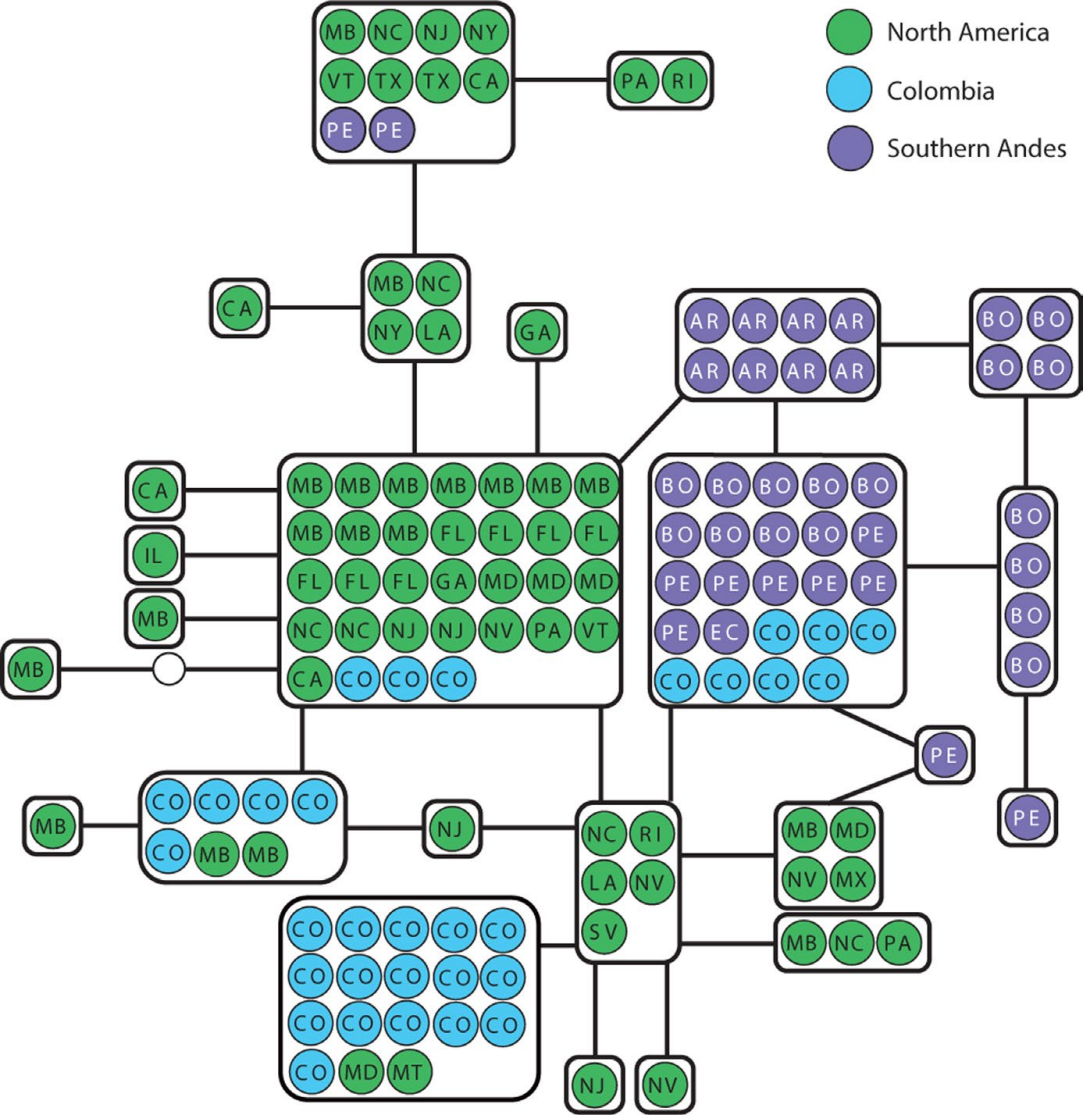
Control region (mtDNA) haplotype network. Populations are indicated by different colors and abbreviations (CO, Colombia; PE, Peru; BO, Bolivia; EC, Ecuador; AR, Argentina). North American localities are abbreviated according to postal codes. Solid lines represent a single mutational change between haplotypes. Hollow circles represent hypothesized unsampled haplotypes

We found a total of 25 mtDNA haplotypes; 15 of these were present only in North America, five occurred only in the Southern Andes, one was shared between North America and the Southern Andes, one was shared between Colombia and the Southern Andes, and three were shared between North America and Colombia (Figure 2). Of the four haplotypes found in Colombia, three (including the most common haplotype in North America) were shared with North America, and the other one was the most common haplotype of the Southern Andes.

**Figure 3:**
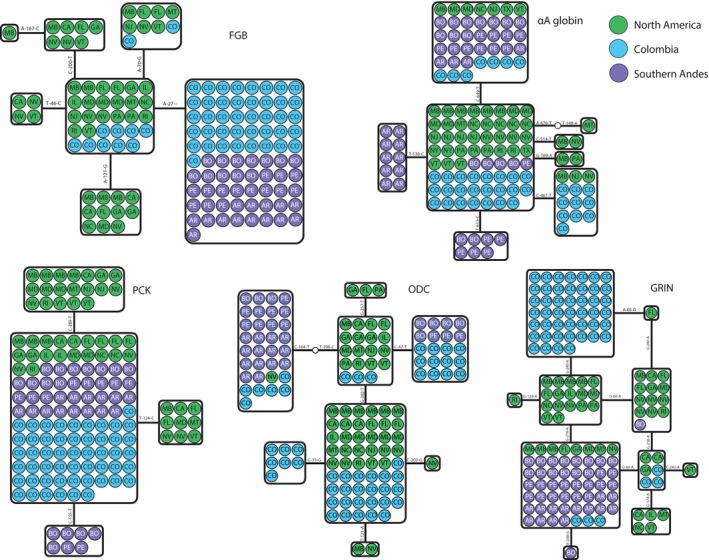
Allelic networks for five nuclear loci (FGB, GRIN1, ODC1, PCK1 and α^A^ globin). Populations are indicated by different colors and abbreviations (CO, Colombia; PE, Peru; BO, Bolivia, EC, Ecuador, AR, Argentina). North American localities are abbreviated according to postal codes. Solid lines represent a single mutational change between haplotypes. Hollow circles represent hypothesized unsampled haplotypes

Varying patterns of genetic variation were observed in nuclear introns (Figure 3). The most common FGB allele was found in the Southern Andes and also occurred at high frequency in Colombia; six different FGB alleles were found in North America, and two of these were found in Colombia as well. For GRIN1, a private allele and a shared allele were found in Colombia, and the most common allele was shared among the three populations. Of the remaining eight GRIN1 alleles, one was private to the Southern Andes, five were private to North America, one was shared between North America and the Southern Andes, and one was shared between North America and Colombia. Two ODC1 alleles were shared between Colombia and the Southern Andes, and between North America and Colombia; three alleles were private to North America, and one was private to Colombia. The most common PCK1 allele was found in all three populations; one additional allele was private to the Southern Andes, and two were private to North America.

We found eight α^A^ globin alleles, three of which were private to North America, two private to the Southern Andes, one was shared between Colombia and North America, and the two most common alleles were shared by the three populations. Allelic richness standardized to the smallest sample size was greater in North America than in Colombia for all but one locus (ODC1), and more allelic diversity was found in Colombia than in the southern Andes for three of five loci (FGB, ODC1, and HBB). However, mtDNA haplotype diversity was greater in the Southern Andes than in Colombia (Table 2).

**Table 2:** Allelic richness corrected to the smallest population simple size (±*SD*) for six loci

Locus	North America		Colombia		Southern Andes	
	*N*	Allelic richness	*n*	Allelic richness	*n*	Allelic richness
mDNA	69	12.2 ± 1.6	31	4	41	8.8 ± 0.9
FGB	50	5.8 ± 0.4	52	3.0 ± 0.0	40	1
GRIN1	48	7.9 ± 0.3	52	3.0 ± 0.0	46	3
ODC1	52	5.2 ± 0.7	52	5.0 ± 0.2	34	2
PCK1	48	3.0 ± 0.0	52	1.0 ± 0.0	40	2
HBB	42	14	50	5.8 ± 0.4	46	2.9 ± 0.3

**Figure 4:**
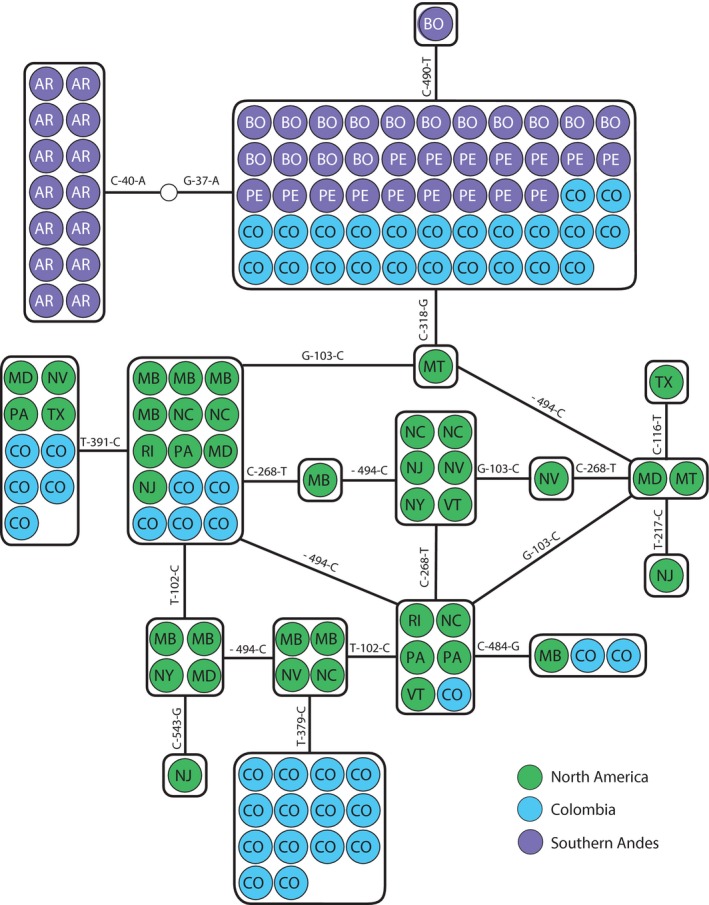
Allelic network for the first 578 bp of the β^A^ globin gene. Populations are indicated by different colors and abbreviations (CO, Colombia; PE, Peru; BO, Bolivia; EC, Ecuador; AR, Argentina). North American localities are abbreviated based on US and Canadian postal codes. Solid lines represent a single mutational change between haplotypes. Hollow circles represent hypothesized unsampled haplotypes

The β^A^ globin showed high levels of variation, with 18 different alleles. The Colombian population exhibited one private allele, one allele shared with the Southern Andes, and four alleles shared with North America. There were 10 alleles private to North America, and two alleles private to the Southern Andes (Figure 4). As with nuclear introns, allelic richness was greater in North America.

**Figure 5:**
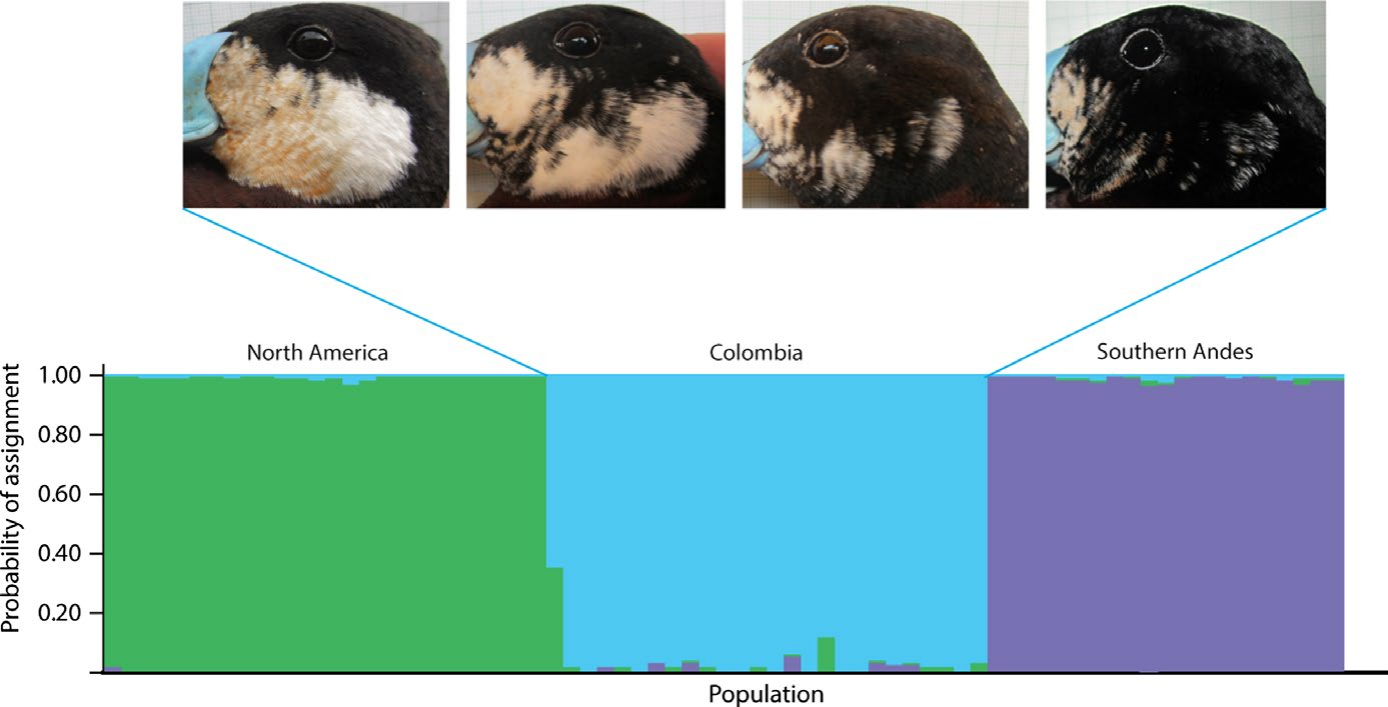
Posterior probability of assignment of individuals to each of the three populations using seven loci. Photos depict lateral views of four adult males sampled at the same lake in Guasca, Colombia, illustrating the marked variation in facial plumage existing within the Colombian population

The STRUCTURE analysis showed strong support for the existence of three genetic clusters (*K *=* *3) corresponding to the three geographic populations of Ruddy Ducks. Most individuals had high probabilities of being assigned (*p* > .93) to their respective population based on their multilocus genotypes, except for two individuals from the Colombian cluster that were genetically admixed with North America (*p* = .60 and *p* = .16; Figure 5). The *H*′ value estimated by CLUMPP was very high (*p* = .99), indicating that all STRUCTURE runs were congruent.

**Figure 6:**
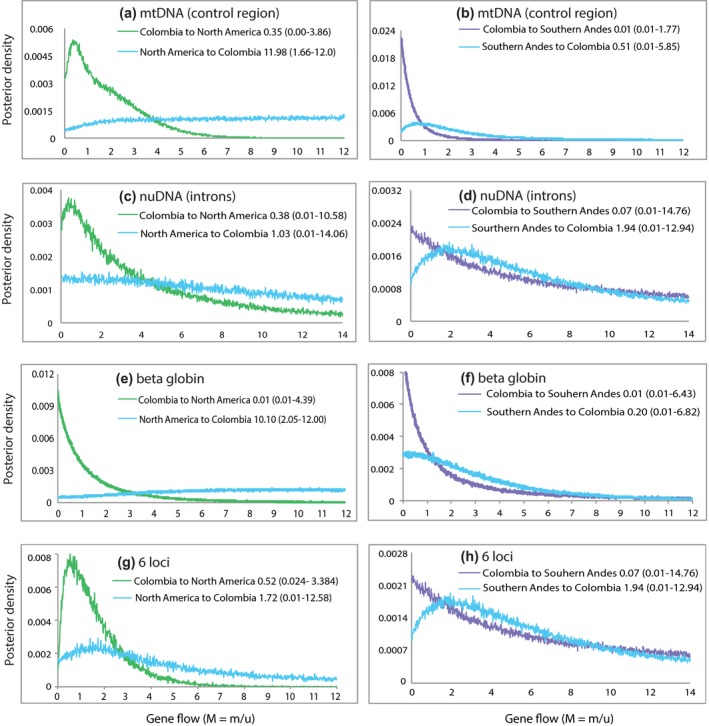
Gene flow estimates for pairwise population comparisons for separate genes (a–f), and all genes combined (g–h). 90% HPDs are shown in parenthesis

Pairwise comparisons of the estimates of gene flow between populations indicated similar patterns in all gene combinations (Figure 6; Table 3). The mtDNA control region revealed the possibility of gene flow from Colombia into North America (*m*/*u* = 0.35), but the posterior distribution overlapped zero, and the posterior gene flow from North America to Colombia was flat, indicating that it could not be estimated reliably (Figure 6a). The mtDNA data also indicated gene flow from the Southern Andes to Colombia (*m*/*u* = 0.51), whereas gene flow on the opposite direction was probably zero (Figure 6b). For nuDNA introns, the peak estimate of gene flow from Colombia to North America was positive (*m*/*u* = 0.38) but the posterior also overlapped zero, and gene flow into Colombia from North America could not be estimated (Figure 6c). Gene flow from the Southern Andes to Colombia estimated based on introns was positive (*m*/*u* = 1.94), but these markers revealed no evidence of gene flow in the opposite direction from Colombia to the Southern Andes (Figure 6d). For the β^A^ hemoglobin, gene flow from North America to Colombia peaked sharply at zero, but it could not be estimated in the other direction (Figure 6e). Hemoglobin gene flow from Colombia to the Southern Andes was also zero, but the posterior distribution of gene flow in the opposite direction was flat (Figure 6f). For all loci combined, estimates of gene flow from Colombia to North America (*m*/*u* = 0.52) and in the opposite direction (*m*/*u* = 1.72) were positive (Figure 6g). In turn, gene flow from Colombia to the Southern Andes appeared to be zero, but gene flow into Colombia from the Southern Andes appeared to have occurred (*m*/*u* = 1.94; Figure 6h).

**Table 3:** IMa2 output of parameter estimates and their respective 90% credible intervals. Effective population size (Θ), splitting time (*t*), splitting parameter (*s*), and migration rate (*M*). Symbol M_1_ > M_2_ symbolizes gene flow from population 1 into population 2

Two‐population IMa2 analysis North America vs. Colombia (mtDNA)							
	ΘN.Am	ΘCol	ΘAncestral	*t*	*s*	MN.Am>Col	MCol>N.Am
High Point	72.075	0.495	0.85	0.8415	0.0045	0.354	11.982
HPD90%Lo	31.725	0.195	0.45	0.3375	0.0155	0.09	0.57
HPD90%Hi	146.325	6.435	90.25	2.9235	0.9625	5.478	11.73

Two‐population IMa2 analysis Colombia vs. Southern Andes (mtDNA)							
	ΘS.Am	ΘCol	ΘAncestral	*t*	*s*	MS.Am>Col	MCol>S.Am
High Point	5.385	1.785	4.05	2.9895	0.0115	0.006	0.512
HPD90%Lo	2.355	0.615	0.75	0.2205	0.0145	0.018	0.138
HPD90%Hi	27.045	16.305	86.95	2.9385	0.9655	3.594	9.282

Two‐population IMa2 analysis North America vs. Colombia (nuDNA)							
	ΘN.Am	ΘCol	ΘAncestral	*t*	*s*	MN.Am>Col	MCol>N.Am
High Point	1.01	0.27	0.869	0.0675	0.0195	0.376	1.032
HPD90%Lo	0.51	0.11	0.275	0.0231	0.0205	0.136	0.296
HPD90%Hi	18.93	16.41	6.413	0.5787	0.9735	14.232	15.368

Two‐population IMa2 analysis Colombia vs. Southern Andes (nuDNA)							
	ΘS.Am	ΘCol	ΘAncestral	*t*	*s*	MN.Am>Col	MCol>S.Am
High Point	0.027	0.31	1.089	0.0369	0.0005	0.072	1.944
HPD90%Lo	0.009	0.13	0.319	0.0195	0.0095	0.184	0.344
HPD90%Hi	1.223	17.39	16.049	0.5811	0.9855	15.304	15.032

Two‐population IMa2 analysis North America vs. Colombia (beta globin)							
	ΘN.Am	ΘCol	ΘAncestral	*t*	*s*	MN.Am>Col	MCol>N.Am
High Point	3.95	0.15	1.375	0.0545	0.9935	0.006	10.098
HPD90%Lo	1.77	0.07	0.187	0.0265	0.0375	0.03	0.594
HPD90%Hi	19.27	9.17	9.053	0.9775	0.9915	7.962	11.742

Two‐population IMa2 analysis Colombia vs. Southern Andes (beta globin)							
	ΘS.Am	ΘCol	ΘAncestral	*t*	*s*	MN.Am>Col	MCol>S.Am
High Point	0.1395	0.73	1.221	0.8075	0.0035	0.006	0.198
HPD90%Lo	0.0645	0.29	0.209	0.0735	0.0095	0.03	0.102
HPD90%Hi	2.4255	13.67	14.861	0.9795	0.9655	10.038	9.846

Two‐population IMa2 analysis North America vs. Colombia (6 loci)							
	ΘN.Am	ΘCol	ΘAncestral	*t*	*s*	MN.Am>Col	MCol>N.Am
High Point	141.075	0.03	12.825	0.1961	0.9995	0.523	1.721
HPD90%Lo	35.925	0.01	4.725	0.0321	0.1565	0.07	0.81
HPD90%Hi	147.975	10.63	35.325	0.1983	0.9925	5.45	895.05

Two‐population IMa2 analysis Colombia vs. Southern Andes (6 loci)							
	ΘS.Am	ΘCol	ΘAncestral	*t*	*s*	MN.Am>Col	MCol>S.Am
High Point	7.275	1.49	5.975	0.9035	0.0325	0.006	1.944
HPD90%Lo	2.595	0.59	1.075	0.1335	0.0155	0.008	0.104
HPD90%Hi	28.455	15.97	35.525	0.9805	0.9635	3.416	10.824

Finally, for the β^A^ hemoglobin, 28% of the Colombian individuals were homozygous for the North American low‐elevation genotype (Thr‐69; Muñoz‐Fuentes et al., 2013), 20% were homozygous for the high‐altitude Andean genotype (Ser‐ 69), and 52% were heterozygous (Thr/Ser‐69).

## 4 DISCUSSION

### 4.1 Biogeography of Ruddy Duck populations

A previous phylogeographic analysis was consistent with the hypothesis that Ruddy Ducks experienced a stepwise colonization of the Northern and Southern Andes from North America, followed by a colonization of high‐to‐low elevations (Muñoz‐Fuentes et al., 2013). However, these inferences were made based on analyses that did not include population‐level sampling from Colombia, the area through which Ruddy Ducks could have entered before expanding in South America, and where genetic adaptations to cope with hypoxia are inferred to have been acquired. If historical occupation indeed occurred north to south, then one should find South American alleles to be derived from North American alleles. If colonization involved genetic adaptations in the hemoglobin gene, then one should find a paraphyletic group formed by lowland alleles from South and North America, with lowland alleles from South America derived from South American highland alleles. Furthermore, if colonization indeed occurred north to south and involved genetic adaptation in the hemoglobin gene, then no or little gene flow from North America into Colombia should be observed in hemoglobin sequences because of the influence of selection, and Colombian individuals should exhibit hemoglobin genotypes previously hypothesized to indicate putative genetic adaptations to life at high elevations (McCracken, Barger, Bulgarella, Johnson, Sonsthagen, et al., 2009; Muñoz‐Fuentes et al., 2013).

Our analyses, the first to include nuclear DNA sequences (i.e., introns, hemoglobin) from Colombia and the first to consider a large number of Colombian individuals sampled for mtDNA, show there is likely more complexity to the historical scenario of north‐to‐south colonization with adaptation to elevation postulated by earlier analyses (Muñoz‐Fuentes et al., 2013). Based on coalescent analyses of multilocus data, gene flow was inferred to occur from the Southern Andes into Colombia and from North America into Colombia. The extent of gene flow from Colombia to North America is difficult to estimate but cannot be rejected, whereas gene flow from Colombia to the Southern Andes was clearly zero (Figure S1). In contrast to the scenario proposed by Muñoz‐Fuentes et al. (2013), these results may be consistent with a south‐to‐north colonization of highland areas from the southern South American lowlands, a pattern seen in different waterfowl species (Fjeldså, 1985; McCracken, Barger, Bulgarella, Johnson, Kuhner, et al., 2009; McCracken, Bulgarella, et al., 2009; Vuilleumier, 1986a; Wilson, Peters, & McCracken, 2012). In addition, these results are consistent with a hypothesis of phenotypic evolution proposed by Siegfried (1976), who considered the black cheek of *ferruginea* to be an ancestral character and the white plumage of *andina* (and *jamaicensis*) to be secondarily developed, because all species except *jamaicenis* and the Eurasian *leucocephala* have black heads. However, because the Colombian population is genetically admixed (see below), with lineages from both North America and the Southern Andes, a plausible interpretation of historical gene flow might be that this population has received immigrants from both the Southern Andes and North America and such gene flow may have obscured our ability to recover the true biogeographic pattern of colonization of different parts of the Americas by Ruddy Ducks. Indeed, because the Colombian population is genetically admixed, we were unable to reliably estimate splitting parameters measuring the fraction of individuals in the ancestral population contributing to one population (Hey, 2005), which would have provided valuable insights on the direction of colonization and allowed for a more direct comparison with results of the earlier study (Muñoz‐Fuentes et al., 2013).

Another discrepancy between our results and those of earlier analyses exists in terms of patterns of variation in the hemoglobin gene. Muñoz‐Fuentes et al. (2013) found a clear association between genotype and elevation when comparing North American and southern South American populations, which led them to propose the hypothesis that colonization of South America via highland environments involved the origin of putative adaptations in hemoglobin function to cope with hypoxia. The validity of this hypothesis was brought into question; however, by functional analyses revealing no discernible effects on oxygen affinity of the variation in hemoglobin sequences between lowland and highland Ruddy Ducks (Natarajan et al., 2015). However, such analyses did not rule out the possibility that genetic variants contributed to variation in other structural or functional properties of the hemoglobin protein (Natarajan et al., 2015). Our results suggest this possibility is unlikely given that we found that not all Colombian individuals have the “high‐elevation” genotype identified by the earlier study: only 20% of the individuals from Colombia were homozygous for the allele previously thought to be restricted to high elevations, 52% were heterozygous, and 28% had the lowland genotype found in North America. This result, together with functional analyses (Natarajan et al., 2015) suggest that a Thr/Ser‐69 substitution arising de novo did not facilitate the colonization of high‐elevation environments by Ruddy Ducks, implying that the previously proposed evolutionary scenario requires reevaluation. Other studies have revealed that patterns of elevational differentiation in hemoglobin sequences consistent with spatially variable selection need not imply adaptive evolution because amino acid replacements may have no effect on respiratory properties of hemoglobin isoforms (Cheviron et al., 2014). However, we note that recent analyses indicate that Ruddy Ducks throughout their range, including North America, possess hemoglobin with relatively high O_2_ affinity, similar to other Andean species (Natarajan et al., 2015). Therefore, high‐affinity hemoglobin might have been preadaptive and facilitated geographic expansion in montane areas even if it did not evolve de novo in the Andes.

Given our results, the broad elevational and latitudinal range of Ruddy Ducks, and the challenges of living at high altitudes, open questions remain regarding the role that evolutionary adaptation or plasticity may have played in the colonization and spread of this species across montane and lowland landscapes. Because adaptive substitutions in the hemoglobin protein arising de novo following colonization of the Andes appear not to have been involved, we suggest that measurements of hematological parameters including hemoglobin concentration and hematocrit would shed light on alternative ways in which Ruddy Ducks may have been able to cope with the challenges of varying partial pressures of oxygen across their extensive range.

### 4.2 Hybrid origin of Colombian Ruddy Ducks?

Our results are consistent with the hypothesis suggested by several authors (Fjeldså, 1986; McCracken & Sorenson, 2005; Siegfried, 1976) that the Colombian population of Ruddy Ducks might be of hybrid origin. Variation in all genes indicated that the Colombian population shares alleles with populations from both North America and the Southern Andes as expected for an admixed population. However, variation in two introns (GRIN1 and ODC) and in the β^A^ globin gene indicates that the Colombian population has likely had enough time to diverge independently and acquire private alleles not found in other populations. In addition, pairwise estimates of genetic structure and STRUCTURE analysis showed that the Colombian population is well differentiated from the others. Furthermore, we observed no relationship between the genetic assignment probability of different individuals and their pattern of cheek coloration (data not shown), which suggests that the phenotypic variability existing among Colombian individuals is not the result of recent influx of individuals from other populations. Taken together, our data may support the hypothesis that during periods of glaciation (Fjeldså, 1986; Van Geel & Van der Hammen, 1973) Ruddy Ducks from the highlands of the Southern Andes could have extended north to the Bogotá plateau in the Colombian Cordillera Oriental, where they may have hybridized with formerly migratory individuals from North America giving rise to a new geographic population which later diverged in isolation following range fragmentation. This hypothesis could be tested more explicitly based on detailed explorations of patterns of genetic variation employing genomic approaches to examine admixture.

## 5 CONCLUSIONS

Our work echoes recent suggestions that examining patterns of genetic and phenotypic (i.e., physiological, plumage) variation with dense sampling in populations from northern South America is critical to gain a comprehensive understanding of the biogeographic history of species with broad distribution ranges in the Americas (Avendaño, Arbeláez‐Cortés, & Cadena, 2017; Pérez‐Emán et al., 2017). Specifically, relative to previous analysis, our addition of samples from the Colombian Andes to phylogeographic analyses revealed (1) that the direction of colonization of Ruddy Ducks appears more difficult to determine—and may even be opposite—than that which was previously inferred, and (2) that if adjustment to high‐elevation conditions played an important role in colonization of new areas by Ruddy Ducks, it likely occurred through different adaptive mechanisms than those previously considered or via phenotypic plasticity in physiological parameters that remain to be studied. In any event, our study also exemplifies the breadth of inferences about species history one can potentially make by combining data from nuclear and functionally important loci in phylogeography (Zamudio et al., 2016).

Finally, we note that the Colombian Ruddy Duck is endemic to isolated high‐Andean wetlands in and it is currently considered endangered (Renjifo, Amaya‐Villarreal, Burbano‐Girón, & Velásquez‐Tibata, 2016; Rosselli & Benítez‐Castañeda, 2016). A recent study highlighted several factors affecting population sizes of endemic and threatened birds living in wetlands of the Bogotá region where the species lives (Rosselli & Stiles, 2012). Increased urbanization, competition with coots, poor water quality, and poaching are the main factors associated with low local abundance. Although there is no evidence of significant declines of the species in the Bogotá area based on Christmas Count data (Stiles, Rosselli, & De La Zerda, 2017), our work provides evidence that the Colombian population of Ruddy Ducks is genetically distinct from other populations of this widespread species. Along with its morphological diagnosability, this population may be considered a full species under different species concepts (Baum & Shaw, 1995; Cracraft, 1983; De Queiroz, 2005, 2007; Templeton, 1989), and minimally, an evolutionary significant unit for conservation purposes (Moritz, 2002).

## CONFLICT OF INTEREST

None declared.

## AUTHOR CONTRIBUTION

MLJ, CDC, and KMC concieved the ideas and designed the methodology. MLJ and KMC conducted fieldwork and collected data. MLJ analyzed the data. MLJ and CDC led the writing of the manuscript. All authors contributed critically to the drafts and gave final approval for publication.
