# Supplementary material for: Neutral and functionally important genes shed light on phylogeography and the history of high‐altitude colonization in a widespread New World duck
Source: Ecol Evol. 2018 Jun 4;8(13):6515–28. doi: 10.1002/ece3.4108 (PMC6053577; doi:10.1002/ece3.4108)
Supplement: Supplementary file 1 [file ECE3-8-6515-s001.pdf]

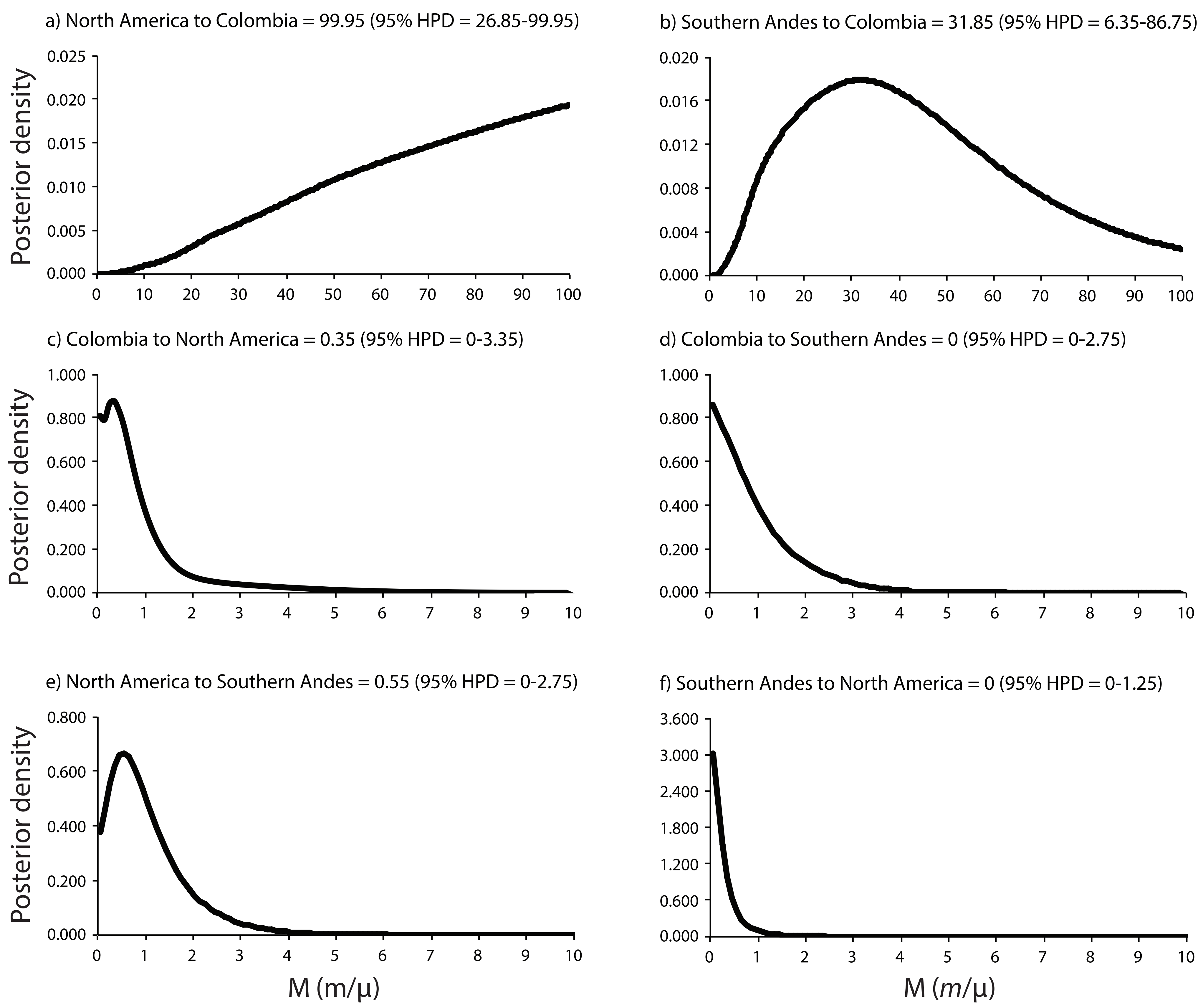

a) North America to Colombia = 99.95 (95% HPD = 26.85-99.95)
b) Southern Andes to Colombia = 31.85 (95% HPD = 6.35-86.75)
c) Colombia to North America = 0.35 (95% HPD = 0-3.35)
d) Colombia to Southern Andes = 0 (95% HPD = 0-2.75)
e) North America to Southern Andes = 0.55 (95% HPD = 0-2.75)
f) Southern Andes to North America = 0 (95% HPD = 0-1.25)
Posterior density
M (m/μ)
M (m/μ)
