# Supplementary material for: Neutral and functionally important genes shed light on phylogeography and the history of high‐altitude colonization in a widespread New World duck
Source: Ecol Evol. 2018 Jun 4;8(13):6515–28. doi: 10.1002/ece3.4108 (PMC6053577; doi:10.1002/ece3.4108)
Supplement: Supplementary file 2 [file ECE3-8-6515-s002.pdf]

Table S1.

Information on samples from Colombia included in phylogeographic analyses and GenBank accession numbers for DNA sequences generated in this study. Blood samples correspond to individuals that were sampled and released, whereas individuals with tissue samples are museum specimens in the ornithological collection of the Museo de Historia Natural de la Universidad de los Andes (ANDES).

						Accession number						
**Catalog No.**	**Locality**	**Latitud**	**Longitud**	**Elevation (m)**	**Sample type**	**Control region**	**PCK1**	**GRIN1**	**FGB**	**ODC1**	**HBA**	**HBB**
OXJA 1	Cundinamarca, Gravilleras de Siecha, Guasca	4.8847	-73.8887	2632	Blood	MH351796	MH351822-3	MH351874-5	MH351926-7	MH351978-9	MH352030-1	MH352070-1
OXJA 2	Cundinamarca, Gravilleras de Siecha, Guasca	4.8847	-73.8887	2632	Blood	MH351797	MH351824-5	MH351876-7	MH351928-9	MH351980-1	MH352032-3	MH352072-3
OXJA 3	Cundinamarca, Gravilleras de Siecha, Guasca	4.8847	-73.8887	2632	Blood	MH351798	MH351826-7	MH351878-9	MH351930-1	MH351982-3	MH352034-5	MH352074-5
OXJA 4	Cundinamarca, Gravilleras de Siecha, Guasca	4.8847	-73.8887	2632	Blood	MH351799	MH351828-9	MH351880-1	MH351932-3	MH351984-5	MH352036-7	MH352076-7
OXJA 5	Cundinamarca, Gravilleras de Siecha, Guasca	4.8847	-73.8887	2632	Blood	MH351800	MH351830-1	MH351882-3	MH351934-5	MH351986-7	MH352038-9	MH352078-9
OXJA 6	Cundinamarca, Gravilleras de Siecha, Guasca	4.8847	-73.8887	2632	Blood	MH351801	MH351832-3	MH351884-5	MH351936-7	MH351988-9	MH352040-1	MH352080-1
OXJA 7	Cundinamarca, Gravilleras de Siecha, Guasca	4.8847	-73.8887	2632	Blood	MH351802	MH351834-5	MH351886-7	MH351938-9	MH351990-1	MH352042-3	MH352082-3
OXJA 8	Cundinamarca, Gravilleras de Siecha, Guasca	4.8729	-73.8893	2641	Blood	MH351803	MH351836-7	MH351888-9	MH351940-1	MH351992-3	MH352044-5	MH352084-5
OXJA 9	Cundinamarca, Gravilleras de Siecha, Guasca	4.8729	-73.8893	2641	Blood	MH351804	MH351838-9	MH351890-1	MH351942-3	MH351994-5	MH352046-7	MH352086-7

						Accession number						
**Catalog No.**	**Locality**	**Latitud**	**Longitud**	**Elevation (m)**	**Sample type**	**Control region**	**PCK1**	**GRIN1**	**FGB**	**ODC1**	**HBA**	**HBB**
OXJA 10	Cundinamarca, Gravilleras de Siecha, Guasca	4.8729	-73.8893	2641	Blood	MH351805	MH351840-1	MH351892-3	MH351944-5	MH351996-7	MH352048-9	MH352088-9
OXJA 11	Cundinamarca, Gravilleras de Siecha, Guasca	4.8729	-73.8893	2641	Blood	MH351806	MH351842-3	MH351894-5	MH351946-7	MH351998-9	MH352050-1	MH352090-1
OXJA 12	Cundinamarca, Gravilleras de Siecha, Guasca	4.8729	-73.8893	2641	Blood	MH351807	MH351844-5	MH351896-7	MH351948-9	MH352000-1	MH352052-3	MH352092-3
OXJA 13	Cundinamarca, Gravilleras de Siecha, Guasca	4.8729	-73.8893	2641	Blood	MH351808	MH351846-7	MH351898-9	MH351950-1	MH352002-3	-	MH352094-5
OXJA 14	Cundinamarca, Gravilleras de Siecha, Guasca	4.8729	-73.8893	2641	Blood	MH351809	MH351848-9	MH351900-1	MH351952-3	MH352004-5	MH352054-5	-
OXJA 15	Cundinamarca, Gravilleras de Siecha, Guasca	4.8729	-73.8893	2641	Blood	MH351810	MH351850-1	MH351902-3	MH351954-5	MH352006-7	-	MH352096-7
OXJA 16	Cundinamarca, Gravilleras de Siecha, Guasca	4.8729	-73.8893	2641	Blood	MH351811	MH351852-3	MH351904-5	MH351956-7	MH352008-9	-	MH352098-9
OXJA 17	Cundinamarca, Gravilleras de Siecha, Guasca	4.8729	-73.8893	2641	Blood	MH351812	MH351854-5	MH351906-7	MH351958-9	MH352010-1	-	MH352100-1
OXJA 18	Cundinamarca, Gravilleras de Siecha, Guasca	4.8729	-73.8893	2641	Blood	MH351813	MH351856-7	MH351908-9	MH351960-1	MH352012-3	MH352056-7	MH352102-3
OXJA 19	Cundinamarca, Gravilleras de Siecha, Guasca	4.8729	-73.8893	2641	Blood	MH351814	MH351858-9	MH351910-1	MH351962-3	MH352014-5	MH352058-9	MH352104-5
OXJA 20	Cundinamarca, Gravilleras de Siecha, Guasca	4.8729	-73.8893	2641	Blood	MH351815	MH351860-1	MH351912-3	MH351964-5	MH352016-7	MH352060-1	MH352106-7
ANDES BT 337	Cundinamarca, Laguna de Fúquene	5.4672	-73.7537	2580	Tissue	MH351816	MH351862-3	MH351914-5	MH351966-7	MH352018-9	-	MH352108-9

						Accession number						
**Catalog No.**	**Locality**	**Latitud**	**Longitud**	**Elevation (m)**	**Sample type**	**Control region**	**PCK1**	**GRIN1**	**FGB**	**ODC1**	**HBA**	**HBB**
ANDES BT 358	Cundinamarca, Laguna de Fúquene	5.4672	-73.7537	2580	Tissue	MH351817	MH351864-5	MH351916-7	MH351968-9	MH352020-1	MH352062-3	MH352110-1
ANDES BT 419	Cundinamarca, Laguna de Fúquene	5.4672	-73.7537	2580	Tissue	MH351818	MH351866-7	MH351918-9	MH351970-1	MH352022-3	-	MH352112-3
ANDES BT 597	Cundinamarca, Laguna de Fúquene	5.4672	-73.7537	2580	Tissue	MH351819	MH351868-9	MH351920-1	MH351972-3	MH352024-5	MH352064-5	MH352114-5
ANDES BT 745	Cundinamarca, Laguna de Fúquene	5.4672	-73.7537	2580	Tissue	MH351820	MH351870-1	MH351922-3	MH351974-5	MH352026-7	MH352066-7	MH352116-7
ANDES BT 1067	Cundinamarca, Laguna de Fúquene	5.4672	-73.7537	2580	Tissue	MH351821	MH351872-3	MH351924-5	MH351976-7	MH352028-9	MH352068-9	MH352118-9
