# Supplementary material for: Neutral and functionally important genes shed light on phylogeography and the history of high‐altitude colonization in a widespread New World duck
Source: Ecol Evol. 2018 Jun 4;8(13):6515–28. doi: 10.1002/ece3.4108 (PMC6053577; doi:10.1002/ece3.4108)
Supplement: Supplementary file 3 [file ECE3-8-6515-s003.pdf]

Table S2.

PCR annealing temperature (Ta) for locus specific primers.

Locus	Primer	Sequence (5´-3´)	Ta
mtDNA (control region)	L78	GTTATTTGGTTATGCATATCGTG	55°C
	H774	CCATATACGCCAACCGTCTC	
ODC1	ODC1-5.F	TCGTTCAAGCCATTTCTGATGCC	62.9°C
	ODC1.6.R	CCAGGRAAGCCACCACCAATRTC	
FGB	FGB-7.F	GTTAGCATTATGAACTGCAAGTAATTG	55.9°C
	FGB-7.R	TTTCTTGAATCTGTAGTTAACCTGATG	
GRIN1	GRIN1-11.F	CTGGTGGGGCTGTCTGTG	55°C
	GRIN1-11a.R	ACTTTGAASCGKCCAAATG	
PCK1	PCK1-9.F	CAGCCATGAGATCTGAAGCA	55.8°C
	PCK1-9.R	TTGAGAGCTGGCTTTCATTG	
HBA	F1	GGGCACCCGTGCTGGGGGCTGCCAAC	70°C
	R366	GCAGCCGCCACCTTCTTGCC	
	F334	GACCTACTTCCCCCACTTTGACC	63°C
	R1rev	CTGGCAACAGGGTGGGTCCAGCTCTAGCC	
HBB	StartF1	GCCACACGCTACCCTCCACCCGACACC	71.3°C
	In2R3Oxy	CCTGCCCATCCTTCTGGATCTGCC	
